# WHO first global patient safety challenge in Hong Kong – successful implementation in a territory-wide healthcare system

**DOI:** 10.1186/1753-6561-5-S6-P319

**Published:** 2011-06-29

**Authors:** CHS Lam, PTY Ching, C Tam, TY Wong, WH Seto, ICNs of HA

**Affiliations:** 1 Hospital Authority, Hong Kong, Hong Kong, China; 2 Centre for Health Protection, Hong Kong, Hong Kong, China

## Introduction / objectives

Hand hygiene is the single most important measure for preventing healthcare associated infection. In Hong Kong the WHO hand hygiene program was launched in a territory wide healthcare system.

## Methods

In 2006, hand hygiene (HH) was piloted in 4 hospitals that included intervention and control wards. The promotion program included education, placement of Alcohol Hand Rub (AHR) at point of care and posters. In 2009, the program was extended to 74 out patient clinics, 14 Tradition Chinese Medicine clinics and 8 long-term Care Facilities.

In 2008, “clean care is safer care” was implemented in 38 public Hospitals. A huge banner sized 9 x 15 meter was posted at the headquarter building. Same design was also put up across all hospitals. New designs were changed annually.

## Results

Compliance rate of control wards was 22.16 % at baseline that increased to 25.27 %. For test wards it was 23.8% that significantly increased to 55.7 % and Chi-square test was <0.05.

For the 74 out patient clinics, 14 Tradition Chinese Medicine clinics and 8 long-term Care Facilities, the overall compliance was 91%, 90% and 80% respectively.

AHR was also measured as a surrogate marker of hand hygiene compliance. The amount used was found to have steadily increased from 3.2 L in 2007 to 12.9 L per 1000 patients bed days in 2010 and the correlation will be done when the data of 2011 is available.

## Conclusion

The WHO Hand Hygiene program can be successfully implemented in different kinds of healthcare by a concerted territory-wide effort.

## Disclosure of interest

None declared.

